# Takotsubo Cardiomyopathy in Traumatic Brain Injury

**DOI:** 10.1007/s12028-016-0334-y

**Published:** 2016-12-20

**Authors:** Chun Fai Cheah, Mario Kofler, Alois Josef Schiefecker, Ronny Beer, Gert Klug, Bettina Pfausler, Raimund Helbok

**Affiliations:** 1 Department of Neurology, Neurological Intensive Care Unit, Medical University of Innsbruck, Anichstrasse 35, 6020 Innsbruck, Austria; 2 Department of Neurology, Hospital Pulau Pinang, Jalan Residensi, 10990 Georgetown, Pulau Pinang Malaysia; 3 University Clinic of Internal Medicine III, Cardiology and Angiology, Medical University of Innsbruck, Anichstrasse 35, 6020 Innsbruck, Austria

**Keywords:** Traumatic brain injury, Myocardial dysfunction, Takotsubo cardiomyopathy, Monitoring, Echocardiography

## Abstract

**Background:**

Takotsubo cardiomyopathy (TC) is a well-known complication after aneurysmal subarachnoid hemorrhage and has been rarely described in patients with traumatic brain injury (TBI).

**Methods:**

Case report and review of literature.

**Results:**

Here, we report a 73-year-old woman with mild traumatic brain injury (TBI) presenting in cardiogenic shock. Takotsubo cardiomyopathy (TC) was diagnosed by repeated echocardiography. Cardiovascular support by inotropic agents led to hemodynamic stabilization after initiation of levosimendan. Cardiac function fully recovered within 21 days. We performed an in-depth literature review and identified 16 reported patients with TBI and TC. Clinical course and characteristics are discussed in the context of our patient.

**Conclusion:**

Takotsubo cardiomyopathy is under-recognized after TBI and may negatively impact outcome if left untreated.

**Electronic supplementary material:**

The online version of this article (doi:10.1007/s12028-016-0334-y) contains supplementary material, which is available to authorized users.

## Introduction

Takotsubo cardiomyopathy (TC) is known to occur in patients with severe brain insult. It has been widely described after subarachnoid hemorrhage (SAH, 1.2–28 %) [1–3]; however, it rarely occurs in patients with intracerebral hemorrhage, ischemic stroke, and traumatic brain injury (TBI) [4]. In medical ICU patients, the incidence ranges between 5.7 and 28 % [5, 6]. Here, we report a case of mild TBI with secondary hematoma progression presenting with severe TC and provide a comprehensive review of all reported TBI cases [7–19].

## Case Report

**Fig. 1:**
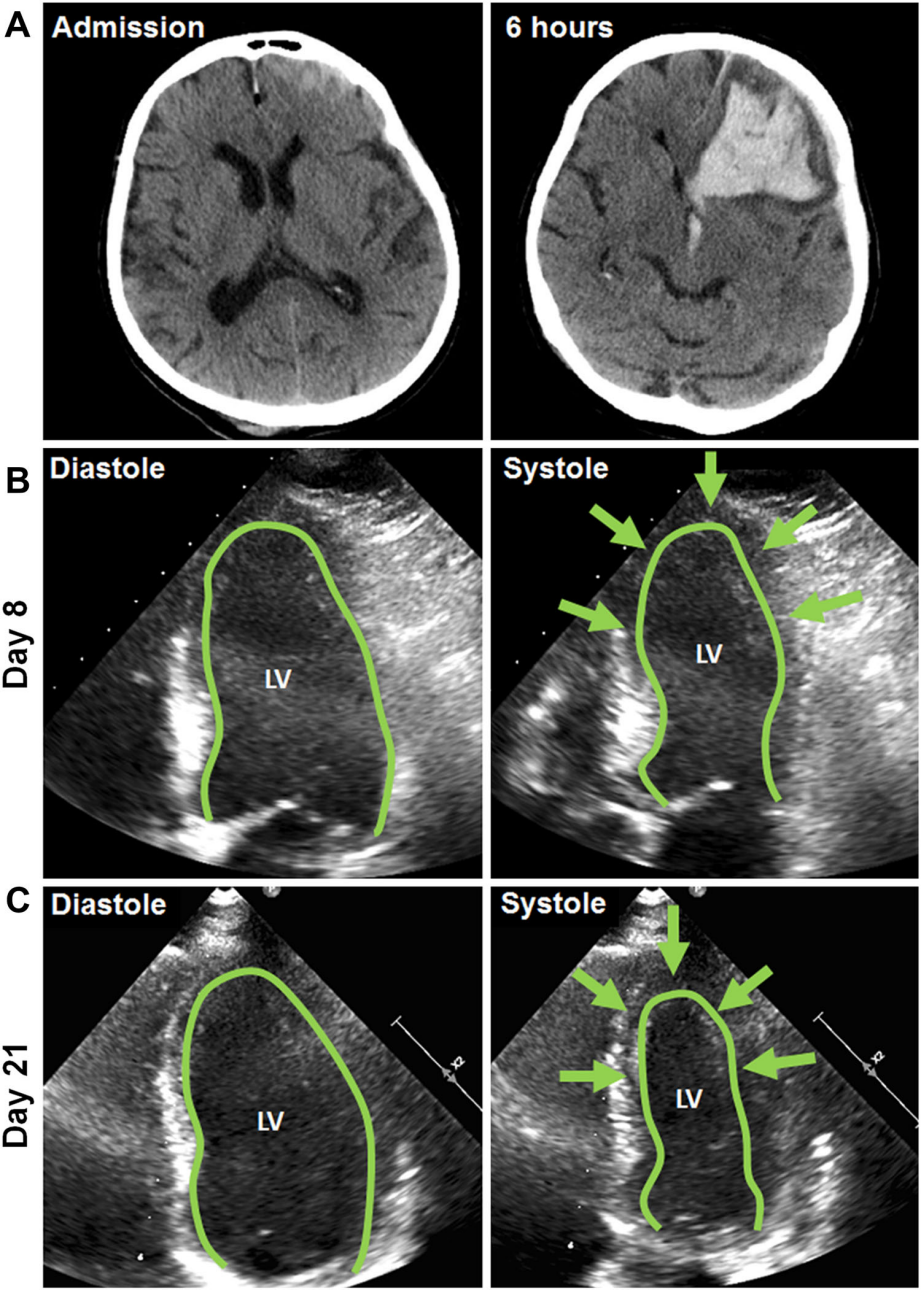
Neuroimaging and echocardiography *Panel A* indicate axial head computed tomography on admission and at follow-up 6 h later. Echocardiography on day 8 indicates moderate reduction in LVEF secondary to a persisting midventricular and apical hypo-/akinesia (*arrows*, *Panel B*). After 21 days (*Panel C*) LVEF nearly normalized and regional wall motion markedly improved (*arrows*) consistent with the typical presentation of a takotsubo cardiomyopathy. *LV* left ventricle, *EF* ejection fraction

A previously healthy 73-year-old woman was admitted to the trauma ward of our tertiary hospital with mild TBI. On presentation, she was disoriented, had a Glasgow Coma Scale (GCS) score of 14, and suffered from retrograde amnesia. Neurological examination revealed bilateral gaze-evoked nystagmus, but no other focal neurological deficit and her vital signs were stable. Laboratory workup revealed 0.21 % blood alcohol concentration. Computed tomography (CT) scanning of the brain showed right parieto-occipital and left temporo-parietal skull fractures with an acute subdural hematoma (ASDH) and traumatic SAH over the left hemisphere and a small left frontal hemorrhagic contusion (Fig. 1, Panel A). Six hours later, the patient deteriorated and repeat head-CT showed a significant progression of the left frontal hemorrhage with intraventricular extension and a midline shift of 11 mm (Fig. 1, Panel A). Hematoma evacuation and placement of an external ventricular drain were immediately performed, and the patient was transferred to the neurological intensive care unit. Postoperatively the patient was on norepinephrine (0.073 mcg/kg/min), sufentanil (0.073 mcg/kg/min), and midazolam (6 mcg/kg/min). Within the next 24 h, norepinephrine had to be continuously increased to 0.29 mcg/kg/min to achieve a cerebral perfusion pressure (CPP) of >65 mmHg. In addition, dobutamine (6.038 mcg/kg/min), phenylephrine (0.725 mcg/kg/min), and hydrocortisone (1.933 mcg/kg/min, given to treat secondary adrenal insufficiency) were necessary to keep the patient hemodynamically stable.

At this time, the electrocardiography showed sinus tachycardia at a rate of 130 beats per minute (bpm) with non-specific repolarization abnormalities with no correspondence to a distinct coronary artery territory. Laboratory myocardial biomarkers exceeded pathologic thresholds: Troponin T levels peaked at 0.54 ng/mL (normal range, <0.014 ng/mL) and NT-proBNP was 4690 ng/L (normal range 0–303 ng/L). Creatinine kinase (CK) was within normal range. Bedside transthoracic echocardiography demonstrated severe left ventricular (LV) myocardial dysfunction (ejection fraction 35 %), marked hypokinesia of the apical and midventricular portions of the left ventricle suggestive of takotsubo cardiomyopathy (TC). Only mild mitral regurgitation was detected. Invasive coronary angiography was not performed because of typical findings on echocardiogram and the limited therapeutic possibility due to intracranial bleeding.

As dobutamine was not improving the severe myocardial dysfunction, levosimendan was added (initial dose 0.03 mcg/kg/min, gradually increased to 0.12 mcg/kg/min) and maintained for 28 h. After initiation, no increased dosage of norepinephrine was needed. The heart rate decreased to less than 100 bpm, dobutamine and phenylephrine could be withdrawn, and norepinephrine was slowly decreased over the following days without significant drops in blood pressure. Repeated transthoracic echocardiography demonstrated improvement in LV myocardial function on day 8 (ejection fraction 40 %) (Fig. 1, Panel B) and further recovery on day 21 (Fig. 1, Panel C, ejection fraction 49.9 %, normal 54–74 %). Coronary angiography was not performed as coronary artery disease deemed unlikely due to recovery in cardiac function in repeated echocardiography suggestive for TC as underlying pathology. The patient was successfully weaned on day 11 and discharged for neurorehabilitation 21 days after trauma. At this time, she was fully awake with a GCS score of 15, mildly disabled with a grade 4 brachio-facial left-sided hemiparesis and dysphagia.

## Review of Literature

### Methods

We performed a comprehensive literature search using the search terms ‘Takotsubo cardiomyopathy,’ ‘Tako-tsubo cardiomyopathy,’ ‘stress cardiomyopathy,’ ‘stunned myocardium,’ ‘transient-left-ventricular ballooning syndrome,’ ‘apical ballooning syndrome,’ ‘myocardial dysfunction’ or ‘heart failure’ together with ‘traumatic brain injury,’ ‘head injury,’ and ‘polytrauma.’ Only articles in English language were included.

## Results

Overall we identified 13 published articles involving 16 TBI patients with TC [7–19] (Table 1). Among these, 13 were adults and 3 of pediatric age. All patients (except 1 uncharacterized) presented with impaired consciousness necessitating mechanical ventilation. The brain injury pattern was heterogeneous including various degrees of contusional hematoma, epidural hemorrhage (EDH), subdural hemorrhage (SDH), and traumatic SAH with 5 patients undergoing neurosurgical intervention. Six patients presented with polytrauma on admission. TC was diagnosed within 24 h in most patients (*N* = 10/16, 63 %); however, one patient developed TC 12 days after admission. In 4 patients, coronary angiography was performed and confirmed TC. Electrocardiography abnormalities were found in 9/16 patients (56 %) including ST segment and *T* wave changes, and 69 % (11/16) had elevated serum troponin levels. Treatment differed; however, most received inotropic support using dobutamine. In one patient, levosimendan at a dose of 0.1 mcg/kg/min was used for 24 h. In addition, various drugs were used to sustain adequate blood pressure including epinephrine, norepinephrine, and vasopressin. Five patients needed extracorporeal life support to treat severe refractory cardiovascular shock. Echocardiography revealed abnormal results in all patients (100 %) and was reversible in the majority of patients within 7 days except in 2 patients after 12 and 17 days, respectively.

**Table 1:** Reported cases of takotsubo cardiomyopathy with traumatic brain injury in the literature

References	Number of patients	Admission level of consciousness, GCS	Imaging	TC onset day	Echocardiography	ECG	Laboratory	Other investigation (s)	Treatment (s)	Functional outcome/cardiac outcome
Palac et al. [7]	1	Unresponsive	tSAH	1	EF 45 % LV hypokinesia sparing apex	NA	CK max = 1244 U/L^a^ Troponin max = 1.4 ng/mL (NR < 0.5 ng/mL)	NA	Dopamine, Norepinephrine, Vasopressin	Mortality: Yes Echo the same day: EF = 60 %
Krishnamoorthy et al. [8]	1	Worsening somnolence	SDH Midline shift	2	EF 35 % Basal hypokinesia	NA	NA	NA	Phenylephrine: 300 mcg Ephedrine: 20 mg Craniectomy	Mortality: No Echo the same day: EF = 55 %
Divekar et al. [9]	1	Unresponsive	SDH	1	EF 45 %. Apical akinesia	T wave inversion in I, aVL, and V4–V6 with QT prolongation	CK max = 853 U/L^a^ Troponin T max = 0.6 ng/mL^a^	CAG: normal	NA	Mortality: NA Echo after 3 days: normal
Deleu et al. [10]	1	GCS 6	Contusion EDH	6	EF 18 %, Diffuse LV akinesia	Sinus tachycardia, diffuse, symmetric T wave inversion	CK max = 311 U/L (NR 39–238 U/L) Troponin max = 0.08 ng/mL (NR < 0.03 ng/mL)	NA	Epinephrine^b^: up to 3 mcg/min Norepinephrine^c^ Craniotomy	Mortality: No Echo after 12 days: EF = 50 %
Wippermann et al. [11]	1	NA	Diffuse edema	1	EF < 10 % LV akinesia	Anterior myocardial ischemia	Troponin I max = 2.3 ng/mL^a^	NA	Inotropes, Craniotomy ECLS	Mortality: Yes Echo after 2 days: EF = 50 %
Maréchaux et al. [12]	1	Impaired consciousness	tSAH	1	EF 20 % LV akinesia sparing apex	Diffuse T wave inversion with QT prolongation	Troponin max = 1.6 ng/mL (NR < 0.1 ng/mL)	NA	NA	Mortality: Yes Echo: NA
Vergez et al. [13]	1	NA	SDH, herniation	2	Severe LV hypokinesia	Marked ST elevation (≥2 mm) negative T waves left precordium	Troponin I max = 3.2 ng/mL (NR < 0.02 ng/mL)	NA	Norepinephrine^c^: 0.–0.83 mcg/kg/min Dobutamine: 15 mcg/kg/min Craniotomy	Mortality: No Echo after 17 days: Improvement in apical LV contractility
Riera et al. [14]	1	GCS 5	Contusion, tSAH	5	Moderate to severe LV hypokinesia sparing apex	Sinus tachycardia, subendocardial injury anteroseptal and inferior	CK max = 242U/L (NR 24–170 U/L) Troponin I max = 1.13 ng/mL (NR < 0.06 ng/mL)	CAG: normal LVG: LV myocardial dysfunction	Norepinephrine: 0.8–1 mcg/kg/min Dobutamine	Mortality: No Echo after 7 days: EF 45–50 %
Samol et al. [15]	1	Comatose	Contusion tSAH	1	LV hypokinesia	T negativity in V3–V6 with QT prolongation	CK max = 480U/L^a^ Troponin I max = 6.8 ng/mL (NR < 0.04 ng/mL)	CAG: normal LVG: LV hypokinesia midventricular Cardiac MRI: severe LV hypokinesia (EF 25 %)	Catecholamines	Mortality: No Echo after 2 days: EF 45 %
Santoro et al. [16]	1	NA	NA	1	EF 30 %	NA	Troponin max = 4.72 ng/mL^a^	CAG: normal	Levosimendan: 0.1 mcg/kg/min	Mortality: No Echo after 3 days: EF 50 %
Krpata et al. [17]	1	GCS 7	Cerebral edema	3	EF 10–15 % LV akinesia	T wave inversion V3–V6	CK max = 541U/L^a^ Troponin max = 3.23 ng/mL^a^	NA	Norepinephrine Milrinone: No dosage given	Mortality: No Echo after 7 days: EF 65 %
Bonacchi et al. [18]	4	NA	Contusion EDH tSAH	1	EF 14 %	NA	NA	NA	Dopamine/dobutamine/epinephrine/norepinephrine/isoproterenol/milnirone: ECLS	Mortality: 2 patients Echo after 3 days: EF 55–59 % in 2 survived patients
Hong et al. [19]	1	GCS 7	tSAH SDH IVH Contusion	12	Moderate LV hypokinesia	Diffuse ST segment elevation in all leads	CK max = 134U/L^a^ Troponin max = 0.11 ng/mL (NR < 0.06 ng/mL)	NA	NA	Mortality: NA Echo after 1 day: Recovered cardiac event

*TBI* traumatic brain injury, *GCS* Glasgow Coma Scale, *tSAH* traumatic subarachnoid hemorrhage, *EDH* extradural hemorrhage, *SDH* subdural hematoma, *IVH* intraventricular hemorrhage, *ECG* electrocardiography, *CAG* coronary artery angiogram, *LV* left ventricle, *LVG* left ventriculogram, *MRI* magnetic resonance imaging, *CK* creatinine kinase, *Max* maximum, *NR* normal range, *EF* ejection fraction, *ECLS* extracorporeal life support, *NA* not available, *echocardiogram* echo

^a^Local laboratory ranges not available

^b^Adrenaline in local institution

^c^Noradrenaline in local institution

In summary, (1) brain injury pattern in TBI patients presenting with TC is heterogeneous and therefore unspecific, (2) in the majority of patients inotropic support using dobutamine leads to improved cardiac function, (3) patients presenting in severe refractory cardiovascular shock may necessitate extracorporeal life support, and (4) with adequate management of TC long-term prognosis is more dependent on the severity of brain injury.

## Discussion

Myocardial dysfunction in various degrees has been reported in patients with brain trauma [20–22] (Table 2), being more prevalent in severe TBI. TC represents a serious manifestation of myocardial dysfunction and is defined as an acute, transient, and reversible heart failure syndrome due to regional wall abnormalities of the ventricular myocardium with associated new electrocardiography changes and elevation of myocardial biomarkers in the absence of culprit atherosclerotic coronary artery disease or cardiac condition causing the temporary ventricular dysfunction [23, 24]. Since its’ initial description in 1990, TC was almost exclusively reported in patients with severe SAH. Only a few reports were published in patients with severe TBI although pathophysiologic mechanisms of both entities may have similar effects on the neuro-cardiac axis. Perhaps, many of the earlier suggested criteria to define TC that excluded the presence of TBI had compounded the conundrum [25].

**Table 2:** Description of 3 studies on myocardial dysfunction in patients with traumatic brain injury

References	Number of patients	Patients severe TBI (%)	Pathology	Abnormal ECG (%)	Increased CK or troponin level (%)^a^	Abnormal echocardiography (%)^b^	Patients with myocardial dysfunction (%)^c^
Bahloul et al. [20]	7	5/7 (85)	EDH, SDH, cerebral edema, contusion	7/7 (100)	2/7 (28.5)	3/7 (42.8)	7/7 (100)
Prathep et al. [21]	139	78/139 (56)	SDH, tSAH contusion	NA	98/139 (30.6)	31/139 (22.3)	31/139 (22.3)
Hasanin et al. [22]	50	50/50 (100)	SDH, tSAH, IVH, DAI, contusion	31/50 (62)	27/50 (54)	21/50 (42)	25/50 (50)

*TBI* traumatic brain injury, *tSAH* traumatic subarachnoid hemorrhage, *ECG* electrocardiography, *EDH* extradural hemorrhage, *SDH* subdural hematoma, *IVH* intraventricular hemorrhage, *DAI* diffuse axonal injury, *CK* creatinine kinase, *NA* not available

^a^According to local laboratory ranges

^b^Evidence for decreased EF or cardiac wall motion abnormality

^c^Clinical presentation and/or echocardiogram evidence of cardiac dysfunction

Underlying pathophysiologic mechanisms are still incompletely understood. Most investigations suggest an interconnected cascade of neuronal injury causing sympathetic overstimulation and direct catecholamine toxicity to the heart [26]. Supra-physiologic levels of epinephrine bind to myocardial B2-receptors causing myocardial protein Gs-to-Gi coupling switch, mediated cyclic adenosine monophosphate (cAMP) calcium overload in myocytes, and contraction-band necrosis reducing cardiac contractility [27, 28].

Our patient had full recovery of cardiac function 21 days after trauma. Even though transient and reversible in nature, some reports suggest recovery even up to 12-week postinjury [23]. Hemodynamic support is critical in patients with severe TBI based on current treatment concepts that emphasize maintenance of an adequate CPP [29]. Improving cardiac function in patients with TC may be achieved by using dobutamine and other pharmacological, or non-pharmacologic treatment including extracorporeal life support. Our patient failed to improve by using dobutamine at a dose of 6.0 mcg/kg/min. After adding levosimendan, cardiac function and heart rate markedly improved.

Recently, the use of levosimendan has been reported in patients with aneurysmal SAH where dobutamine was deemed ineffective [30]. Levosimendan is a non-catecholamine inodilator used in the treatment of acute heart failure with higher improvement rate in cardiac function compared to dobutamine [31]. It increases the sensitivity of myofilaments to calcium, leading to increased myocardial contraction without increasing intracellular cAMP or calcium concentrations [31]. Through the opening of an ATP-dependent potassium channel, vasodilatory effects in systemic, coronary, pulmonary, and venous blood vessels may be observed [31]. Unlike other vasopressors, it improves myocardial contractility without increasing myocardial oxygen consumption, and more importantly its action is independent of interactions with adrenergic receptors [31]. Nonetheless, its utilization in patients with TC remains scarce, bearing the rarity of the entity itself. In one of the largest case series, levosimendan was successfully used in 13 patients with TC [16].

## Conclusions

We highlight the presentation of a patient suffering from TBI with takotsubo cardiomyopathy. Although transient in nature and commonly associated with a good overall prognosis, increasing evidence suggests it is a more serious acute cardiac disorder with a variety of complications [23, 24]. Its hemodynamic effect may be deleterious in certain TBI patients if unrecognized. Levosimendan may be an effective therapeutic agent in severe cases.

## Electronic supplementary material

Below is the link to the electronic supplementary material.

Supplementary material 1 (AVI 3633 kb)

Supplementary material 2 (AVI 3492 kb)
